# Pharmacists working in residential aged care: a survey of pharmacist interest and perceived preparedness

**DOI:** 10.1007/s11096-023-01686-7

**Published:** 2024-02-05

**Authors:** Amanda J. Cross, Deborah Hawthorne, Lisa Kouladjian O’Donnell, Kenneth Lee, Amy Theresa Page

**Affiliations:** 1 Centre for Medicine Use and Safety, Faculty of Pharmacy and Pharmaceutical Sciences, Monash University, 381 Royal Parade, Parkville, VIC 3052 Australia; 2 Western Australian Centre for Health & Ageing, School of Allied Health, University of Western Australia, Perth, Australia; 3 Laboratory of Ageing and Pharmacology, Kolling Institute, Faculty of Medicine and Health, The University of Sydney and the Northern Sydney Local Health District, Sydney, Australia; 4 Centre for Optimisation of Medicines, School of Allied Health, University of Western Australia, Crawley, Australia

**Keywords:** Aged, Employment, Homes for the Aged, Inappropriate prescribing, Medication therapy management, Pharmacists, Surveys and Questionnaires

## Abstract

**Background:**

Pharmacists involvement in residential aged care facilities has traditionally been limited to that of an external contractor providing medication reviews, or medication supply.

**Aim:**

To explore Australian pharmacists’ interest and perceived preparedness to work as on-site pharmacists in residential aged care.

**Method:**

National cross-sectional anonymous online survey open for two weeks (September 17th to October 1st 2022) consisting of Likert-type, multiple choice and multiple selection questions. Australian pharmacists were recruited using a broad advertising strategy which included social and traditional media platforms, and snowball sampling. Data were collected on pharmacist self-reported interest and perceived preparedness to work as on-site aged care pharmacists in residential aged care. Data were analysed using descriptive statistics.

**Results:**

Responses were received from 720 participants, 643 were eligible. Most participants were female (n = 466, 73%) and mean (SD) age was 43.5 (SD 12.5) years. Over half the participants were interested or extremely interested in working as an on-site aged care pharmacist (56%, n = 360), and agreed or strongly agreed (n = 475, 76%) that they felt prepared to work as an on-site aged care pharmacist. Most pharmacists felt prepared to engage in a variety of roles within the facilities (> 73% for each role), including resident and system level roles, and the majority agreed they felt prepared to engage with stakeholders, including general practitioners (93%) and medical specialists (86%).

**Conclusion:**

Pharmacists reported they are interested and feel prepared to work as on-site aged care pharmacists. These findings will inform the roll-out of this new model of care to enhance multidisciplinary collaboration in residential aged care.

**Graphical Abstract:**

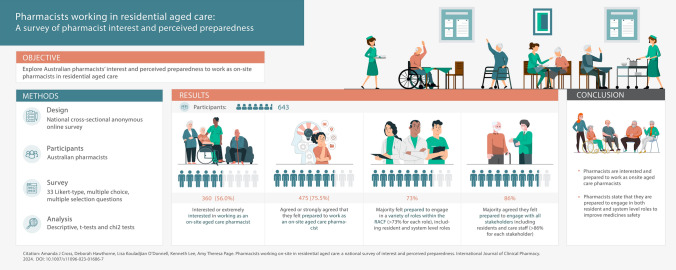

**Supplementary Information:**

The online version contains supplementary material available at 10.1007/s11096-023-01686-7.

## Impact statements

- This study helps to understand the broad pharmacy workforce’s self-reported interest and preparedness to work in residential aged care.
- Findings can be used to inform the roll-out of the Australian Government’s on-site aged care pharmacist model commencing in 2024.
- Pharmacists with practice experience in aged care or conducting medication reviews felt more interested and prepared to work in aged care, and may be prioritized for positions during early roll-out.
- These findings may help to identify training needs of pharmacists moving into aged care pharmacist roles, including training on clinical governance.

## Introduction

Medication safety represents a major issue in residential aged care facilities. Over 95% of people living in residential aged care have at least one medication-related problem detected at the time of a medication review, and over half of all residents are prescribed potentially inappropriate medications [1]. New workforce models are required to address suboptimal medication use and reduce avoidable adverse medication-related events.

Pharmacist involvement in residential aged care can improve medication appropriateness [2], reduce polypharmacy [3] and reduce falls rates [4]. In the US, direct involvement of pharmacists is strongly encouraged to decrease the use of high-risk medications, such as antipsychotics, hypnotics and anxiolytics [5]. However, pharmacist involvement in residential aged care has traditionally been restricted to medication supply or medication review provided by an external contracted pharmacist [6]. This is despite strong support for further involvement of pharmacists in aged care [7]. In Australia, pharmacist involvement mostly consists of community pharmacists supplying medication and dose administration aids, and consultant pharmacists conducting comprehensive medication reviews, known as residential medication management reviews (RMMRs). RMMRs are conducted upon referral from a resident’s general practitioner (GP). Even though the benefits of medication review are widely known, less than 20% of residents receive an RMMR within three months of entering residential aged care, and only 43% receive a review within 12 months [8].

There is growing evidence to support a model of care involving embedded on-site pharmacists in residential aged care [9, 10]. In the UK, the Medicines Optimisation in Care Homes MOCH) program involving onsite clinical pharmacists has led to reduction in polypharmacy, hospital admissions and drug costs [11, 12]. An Australian pilot study reported that on-site aged care pharmacist interventions halved the likelihood of residents being prescribed a potentially inappropriate medication [9]. Following the success of international trials and Australian pilot studies, the Australian Government is investing $345.7 million in a phased implementation of on-site embedded pharmacists in residential aged care [13, 14]. This initiative is expected to require approximately 800 full-time equivalent pharmacists by 2026. As pharmacists move into this new embedded role, there is a need for workforce planning to ensure there are sufficient pharmacists who are appropriately trained and suited for this role. To date, there has been no comprehensive consultation with the Australian pharmacist workforce, nor any international workforce, to determine the level of interest and perceived preparedness of pharmacists to work as on-site pharmacists in residential aged care.

## Aim

This study aimed to explore Australian pharmacists’ interest and perceived preparedness to work as on-site pharmacists in residential aged care.

## Ethics approval

Ethical approval for this study was granted by the Monash University Human Research Ethics Office (Ethics ID 35785, approval 16th September 2022).

## Method

A national anonymous cross-sectional open online survey was utilised. The survey development and recruitment have been reported previously in more depth [15], and described briefly here. This manuscript has been reported as per the Checklist for Reporting of Survey Studies (CROSS) [16].

### Questionnaire development

Questions and response items were developed by the research team (AJC, DH, LKO, KL, ATP). All members of the research team were registered and practicing consultant pharmacists with extensive research experience in questionnaire development, validation, quantitative and qualitative analysis. Consultant pharmacists, also known as accredited pharmacists in Australia, have received post-registration certification and are specially trained to conduct medication reviews.

There were a total of 33 quantitative questions including; two eligibility, five demographic, five related to education and practice experience and 21 Likert-type questions relating to interest and perceived preparedness to work as an on-site aged care pharmacist in residential aged care. Study data were collected and managed using REDCap electronic data capture tool hosted and managed by Helix (Monash University) [17].

### Participants and sample size

The target population for this study was Australian pharmacists, defined as pharmacists registered with the Australian Health Practitioner Regulation Authority (AHPRA). Extrapolating from the challenges described from conducting surveys with general practitioners [18], a convenience sample methodology was chosen. The survey was open for a two-week period (September 17th to October 1st 2022), and aimed to recruit as many pharmacists as possible in the time period.

### Recruitment

Participants were recruited using a broad advertising strategy, which included requests for advertising/broadcasting the study on social and traditional media platforms and direct contact of pharmacists known to the research team to further disseminate to their pharmacist teams. No incentives were offered for participating in the survey. The link to the survey was included in each advertisement and an open survey approach so that participants did not have to register to complete the survey.

### Data analysis

All responses where respondents were eligible, consented and answered the minimum question set (demographics, education and experience) were analysed. Completion rates (ratio of participants who completed minimum question set to those who completed the questionnaire) were calculated. Duplicates were removed comparing demographics using the ‘duplicates’ command in STATA and having potential duplicates reviewed by at least two authors. No cut-point for questionnaires with atypical timestamps was applied, and IP addresses were not tracked.

All statistical analyses were conducted using Stata (StataCorp. 2019. Stata Statistical Software: Release 16. College Station, TX, USA: StataCorp LLC). Figures were prepared using the R Statistical language (version 4.2.1; R Core Team, 2022), using the packages Likert (version 2.0.0; Bryer J, 2022) and ggplot2 (version 3.3.6; Wickham H, 2016). Descriptive statistics were used to describe demographics and Likert-question answers: frequencies and percentages were reported for categorical and ordinal data, and mean and standard deviation were reported for continuous data.

Bivariate analyses, to compare demographics of pharmacists with interest and preparedness, were performed using Pearson’s χ^2^ and one way analysis of variance (ANOVA). Any question answered on a five-point Likert scale that had fewer than five participants indicate the same response were collapsed to a three point Likert scale during bivariate analyses (e.g. combining strongly agree and agree to agree, and similarly combining strongly disagree and disagree) to ensure adequate sample size for analyses and to preserve participant anonymity. A *p*-value of < 0.05 was considered statistically significant.

## Results

### Completion rate

Responses were received by 720 participants who were eligible and consented to participate. Seventy-three participants did not complete the minimum question set (demographics, education and experience) and four responses were identified as duplicates and removed. This left a total 643 participants who responded to the minimum question set (basic demographics, education, accreditation), and 582 (90.5%) completed all questions.

### Demographics

Of the 643 participants, 72.5% (n = 466) were female, the mean (SD) age was 43.5 (12.5) years. The mean (SD) years since first registering as a pharmacist was 18.6 (13.3) years, and 208 (32.3%) were early career pharmacists (≤ 10 years practicing). More than half of the participants worked in metropolitan setting (n = 414, 64.4%), and the most common state was Victoria (n = 212, 33.0%). More than half (n = 363, 56.5%) had completed additional formal qualifications beyond their Bachelor/Master of Pharmacy, and 57.7% (n = 370) were consultant pharmacists. The most common main roles were community pharmacy (n = 266, 41.4%), hospital pharmacy (n = 173, 26.9%), conducting home medication reviews (HMRs) (n = 115, 17.9%) and working in aged care (conducting RMMRs and/or being embedded, n = 106, 16.5%). Twenty-nine (4.5%) participants had experience as an embedded aged care pharmacist, with 15 (2.3%) specifying it as their main role. Full demographics are presented in Table 1.

**Table 1:** Demographics, education and experience of survey participants

Characteristic	Participants, n (%) unless stated
Age, mean (SD)	43.5 (12.5)
Years since registering as a pharmacist, mean (SD)	18.6 (13.3)
Gender	
Man	163 (25.3)
Woman	466 (72.5)
Non-binary/gender diverse	5 (0.8)
Prefer not to say	9 (1.4)
Australian State/Territory	
Australian Capital Territory	22 (3.4)
New South Wales	137 (21.3)
Northern Territory	7 (1.1)
Queensland	108 (16.8)
South Australia	64 (10.0)
Tasmania	26 (4.0)
Victoria	212 (33.0)
Western Australia	63 (9.8)
Prefer not to say	4 (0.6)
Geographical setting, not mutually exclusive	
Metropolitan	414 (64.4)
Regional	217 (33.7)
Rural	99 (15.4)
Remote	17 (2.6)
Consultant pharmacist	
Yes	370 (57.7)
No	271 (42.3)
Additional highest Qualifications*	
None	261 (40.7)
Bachelor/Honours	56 (8.7)
Graduate Certificate	111 (17.3)
Graduate Diploma	68 (10.6)
Masters	96 (15.0)
PhD	32 (5.0)
Prefer not to answer	18 (2.8)
Practice Experience, n (%), not mutually exclusive	
Community	546 (84.9)
Hospital	350 (54.4)
GP Clinic	67 (10.4)
HMR	296 (46.0)
Aged care (RMMR/embedded)	229 (35.6)
Research/Academia	126 (19.6)
Other#	116 (19.6)
Main Role, n (%), not mutually exclusive	
Community	266 (41.4)
Hospital	173 (26.9)
GP Clinic	19 (3.0)
HMR	115 (17.9)
Aged care (RMMRs/embedded)	106 (16.5)
Research/Academia	35 (5.4)
Other#	52 (8.1)

*Summarised to qualification levels, # includes industry, government, regulation, policy and other options provided by participants as free text

*GP* General Practice, *HMR* Home Medicines Review, *PhD* Doctor of Philosophy, *RMMR* Residential Medication Management Review, *SD* standard deviation

### Interest

**Fig. 1:**
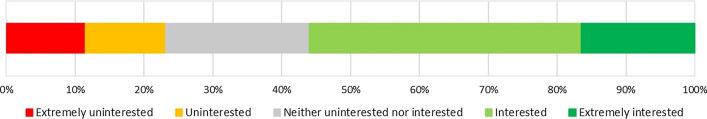
Pharmacists’ interest in working as an on-site aged care pharmacist. Participants n = 643

More than half the participants (n = 360, 56%) were interested or extremely interested in working as an on-site aged care pharmacist, compared to 23% (n = 149) who were uninterested or extremely uninterested (Fig. 1). In bivariate analyses, consultant pharmacists, pharmacists with less years of practice experience who had practice experience conducting HMRs, practice experience in aged care, current main role in hospital and current main role in aged care were significantly associated with being interested in working as an on-site aged care pharmacist (Supplementary Table 1).

### Preparedness to work as an on-site aged care pharmacist

**Fig. 2:**
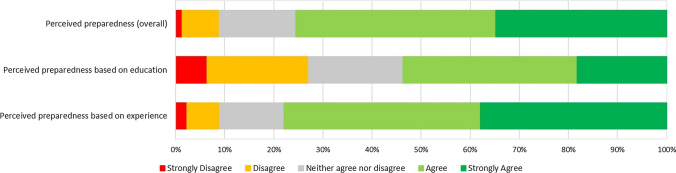
Pharmacists’ perceived preparedness to work as an on-site aged care pharmacist. Participants: Preparedness (overall) n = 629, preparedness based on education n = 643, preparedness based on experience n = 643

More than three quarters of participants (76%, n = 475) felt prepared to work as an on-site embedded pharmacist (Fig. 2). In bivariate analyses, consultant pharmacists, having hospital practice experience, HMR practice experience, aged care practice experience, a current main role not in community pharmacy, a current main role in hospital, and a current main role in aged care were significantly associated with pharmacists agreeing they felt prepared for a role as an on-site aged care pharmacist (Supplementary Table 2).

There was a significant association between interest and preparedness (*p* < 0.001), with 285 (44.3%) of participants agreeing or strongly agreeing that they were both interested and prepared to work as an on-site aged care pharmacist.

About half the participants (n = 345, 54%) agreed or strongly agreed that their formal education had prepared them to work as an on-site aged care pharmacist, and more than a quarter (n = 174, 27%) disagreed or strongly disagreed (Fig. 2). In bivariate analyses, no significant association was found between highest level of qualification and preparedness (Supplementary Table 3).

Of 643 participants, 78% (n = 501) agreed or strongly agreed that their practice experience had prepared them to work as an on-site aged care pharmacist, compared to only 9% (n = 58) who disagreed or strongly disagreed (Fig. 2). In bivariate analyses, consultant pharmacists, having hospital practice experience, HMR practice experience, aged care practice experience, a current main role not in community pharmacy, a current main role in hospital, and a current main role in aged care were significantly associated with pharmacists agreeing they felt prepared for a role as an on-site aged care pharmacist (Supplementary Table 4).

### Preparedness for key activities of an on-site aged care pharmacist

**Fig. 3:**
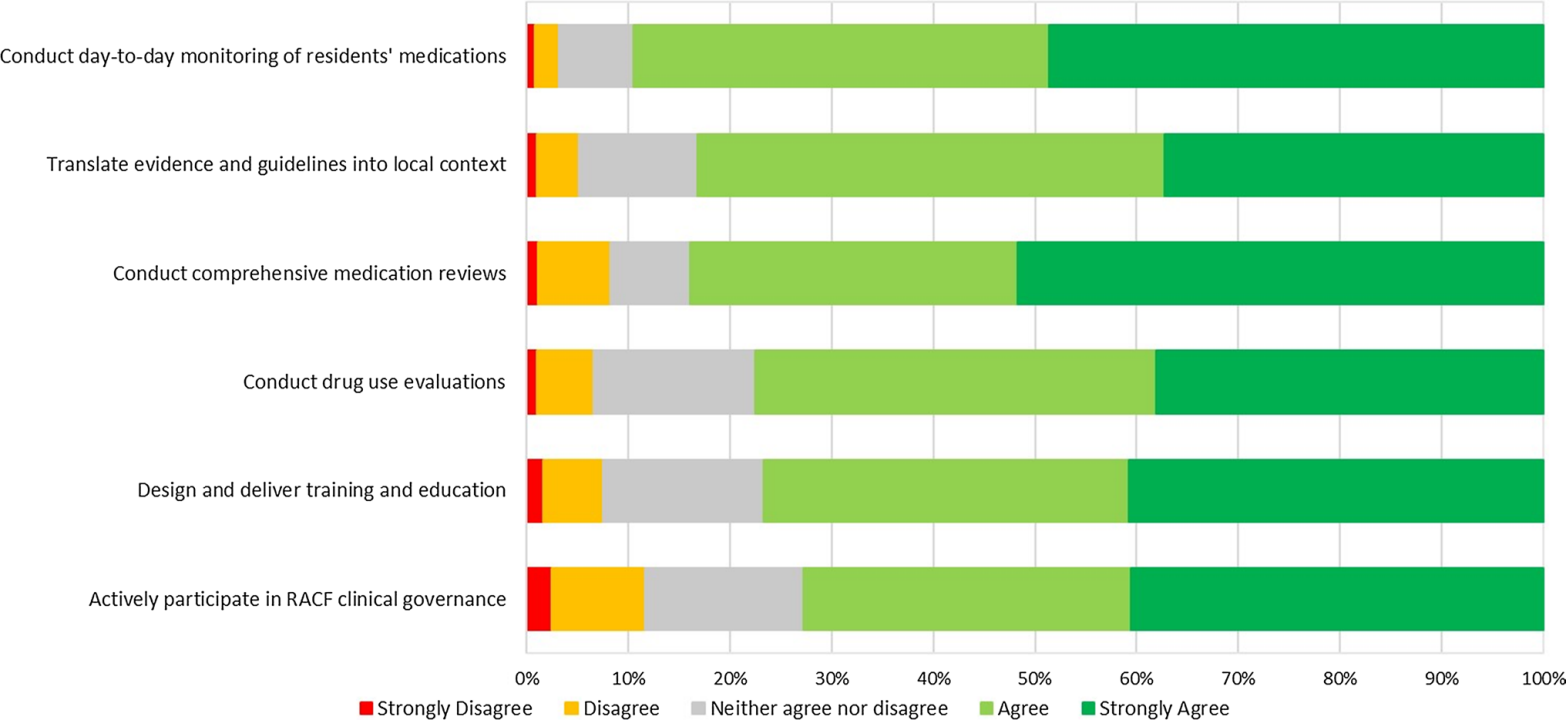
Pharmacists’ perceived preparedness to undertake activities of on-site aged care pharmacist. Participants (n = 609)

The majority of pharmacists agreed they were prepared to conduct the key activities of an on-site aged care pharmacist, ranging between 73 and 89% for each role (see Fig. 3). The activity that pharmacists felt most unprepared for (i.e. answered strongly disagree or disagree) was ‘actively participate in RACF [residential aged care facility] clinical governance, including Medication Advisory Committees’ (n = 72, 12%).

**Fig. 4:**
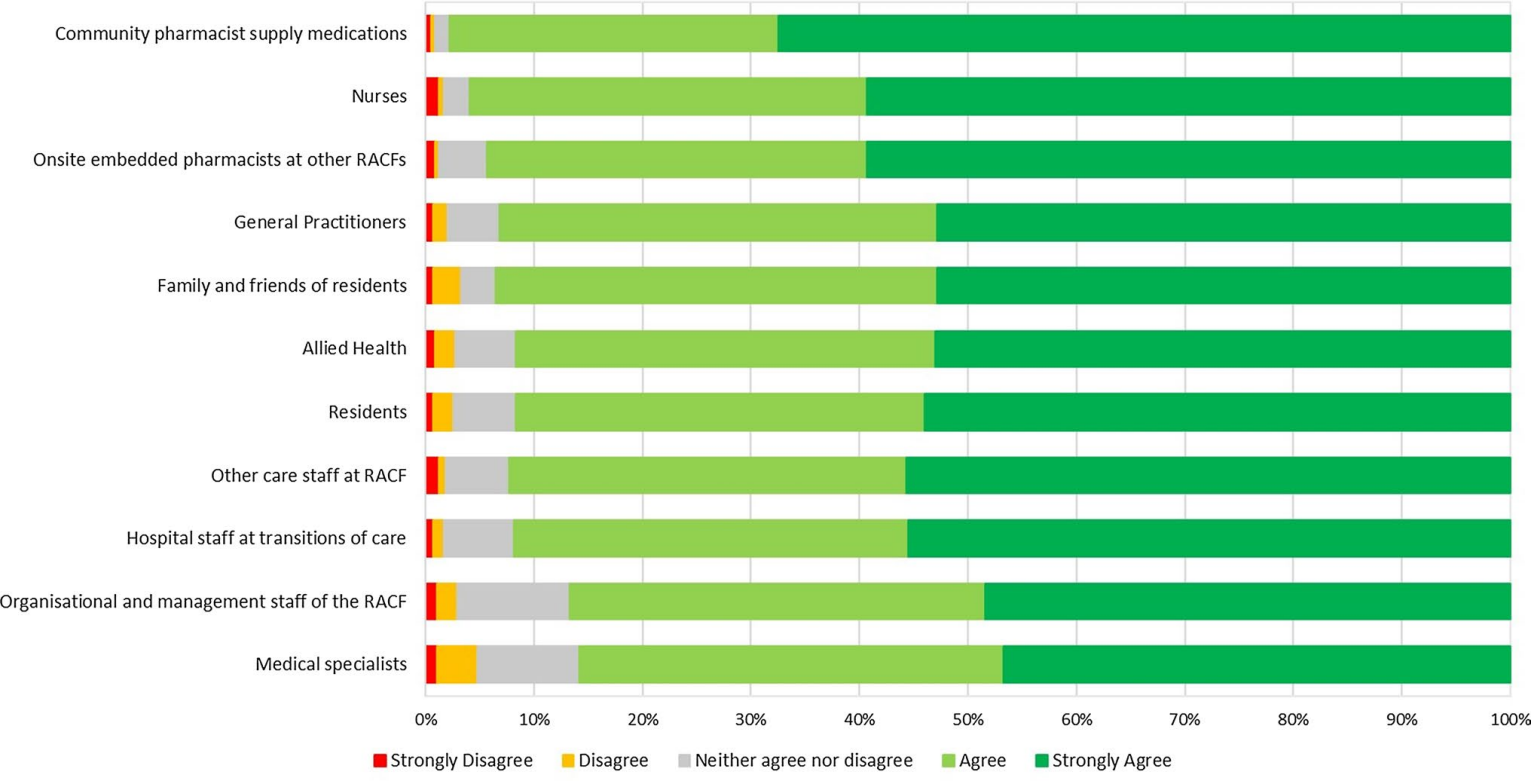
Pharmacists’ perceived preparedness to engage with different stakeholders in residential aged care that aim to improve quality use of medications for residents. Participants (n = 587)

The majority of participants agreed that they felt prepared to engage with stakeholders, ranging from 86 to 98% for each stakeholder (Fig. 4). Most pharmacists felt prepared to engage with general practitioners (n = 545, 93%) and medical specialists (n = 504, 86%).

## Discussion

Our results found that more than half of all participants were interested and three-quarters reported that they felt prepared to work as on-site aged care pharmacists. This large survey is the first study that explored a national pharmacist workforces’ interest and preparedness to work as on-site aged care pharmacists.

Almost 300 pharmacists reported they were both interested and felt prepared to work as on-site pharmacists in residential aged care. Given this survey captured responses from 2% of the Australian pharmacist workforce, it suggests there is enough interest in the role to fill the aged care workforce requirements for the first year of the proposed four-year phased implementation (> 30% of ~ 800 full time equivalent pharmacists). Although, effective workforce planning will be required to avoid depleting pharmacists from other settings (e.g. hospital and community) and to ensure equitable provision of pharmacist services in regional, rural and remote areas compared to metropolitan areas [15]. Resources and support from professional organisations, aged care providers and the Australian Government will also be critical to supporting the existing and emerging workforce in this setting [15, 19].

The majority of pharmacists perceived that their experience prepared them for working as an on-site aged care pharmacist compared to just over half who perceived that their formal education had prepared them. This highlights the importance of practice experience in pharmacist readiness, and parallels the findings from analysis of the free-text questions [15]. Key factors influencing preparedness included familiarity with the aged care setting, resident-level clinical skills obtained through both qualifications and experience, competencies in communication and team work, and direct and indirect experience with system-level quality use of medication activities [15]. Greater integration of skills and competencies required to be an aged care pharmacist into pharmacy university curriculum may be necessary as part of workforce planning, and to reduce the onus on individual pharmacists to participate in extensive postgraduate training. In the United States, an experiential and didactic learning experience for second-year pharmacy students, which included weekly visits to a residential aged care facility, enhanced student geriatric pharmacy knowledge [20]. In the UK’s MOCH, the on-site aged care pharmacists participated in an 18-month training pathway (including pharmacist prescribing training) [21]. Health Education England commissioned the Centre for Pharmacy Postgraduate Education to provide the training pathway to 600 pharmacists [21]. In Australia, it is likely pharmacists will need to be credentialed through an Australian Pharmacy Council approved accredited pharmacist training provider [22]. It remains unclear if this training pathway will be integrated into undergraduate courses or solely provided as a postgraduate specialisation at the financial expense of individual registered pharmacists. Regardless, it will be important to retain the aged care expertise of the existing consultant pharmacist workforce, thus Governments and policy makers designing the on-site pharmacist model need to ensure it is a satisfying and viable career pathway for pharmacists, including experienced consultant pharmacists.

Our study demonstrates that pharmacists felt prepared to engage in both resident- and system-level roles and collaborate with multidisciplinary stakeholders to ensure quality use of medications. This is consistent with the integrated resident and system-level interventions described by Australia’s earliest adopters of the on-site aged care pharmacist model [10]. Being on-site, the pharmacist has greater opportunity to work with the resident and their families and serve as an advocate to ensure their medication use in line with their personal goals of care [10, 23]. Effective collaboration and communication with healthcare professionals and aged care management staff is also important in driving pharmacist-led interventions in residential aged care, such as antimicrobial stewardships [9, 24]. Acting as a linkage agent between stakeholders can facilitate more effective translation of evidence and guidelines into practice [25]. In Australia there are a growing number of pharmacists in other embedded roles, such as General Practice Pharmacists [26, 27]. Internationally pharmacists are increasingly practicing to their full scope as members of primary healthcare teams including in Department of Defence and Veterans Administration [28]. Drawing on learnings from these settings, the relationships with, and support from, medical practitioners and residential aged care staff will be critical to the roll out of this new role [29].

### Strengths and limitations

Our study has several strengths and limitations. Strengths include the large sample size that is broadly representative of the pharmacist workforce in terms of geography and practice experience [30]. Our sample had a higher proportion of women, was older, and had more years of practice experience than the average Australian pharmacist, although these demographics mirror those of consultant pharmacists in a recent study [31] and may have been linked to the overrepresentation of consultant pharmacists in our sample. More than half of the respondents were consultant pharmacists, but this was not unexpected given they are the likely initial target audience for this emerging role. Lack of clarity regarding the specifics of the on-site pharmacist role has been reported as a key factor influencing pharmacists’ current level of interest [15], and likely contributed to the moderate proportion of people who were neither interested nor uninterested. A further limitation of the broad advertising strategy used to recruit participants is that there was a risk of receiving non-pharmacist responses. This risk was mitigated by two members of the author team assessing potential inconsistencies in demographic, education and practice experience responses. Due to the anonymous nature of the survey it was not possible to ask pharmacists for proof they were pharmacists (e.g. AHPRA registration number). In addition, a limitation is that our survey involved a self-reported questionnaire and we could not measure empirically or objectively whether pharmacists were prepared enough to manage this type of role in practice.

### Interpretation and implications

This study provides the first understanding of pharmacist self-reported interest and preparedness to work in residential aged care from the broader pharmacy workforce. Given pharmacists with practice experience in aged care or conducting medication reviews felt more interested and prepared than those without that experience means workforce planning for the roll-out should consider prioritising those pharmacists for early-adopter positions. When further details of the on-site aged care pharmacist model are available, future research could seek to identify if clarity on the model increases pharmacists’ level of interest, particularly among pharmacists who have no aged care or medication review experience. Further research could also seek to confirm whether self-reported preparedness prior to commencing in an on-site aged care role, correlates with actual preparedness once acting in the role.

In preparing the wider workforce and long-term planning, training providers should consider the elements of the role that pharmacists felt least prepared for, including driving quality use of medications at a system-level such as engaging with or leading clinical governance committees (e.g. Medication Advisory Committees). These system-level interventions have been an integral component of the role of early-adopters of the on-site aged care pharmacist model [10], and are included in the accreditation standards for aged care pharmacist education programs [22]. In Switzerland, nursing homes with clinical pharmacists are more likely to have system-level structures and processes related to medication use and safety, than nursing homes without pharmacists [32]. Sharing of knowledge between aged care pharmacists [10], as well as establishing frameworks for transnational collaboration [33] will be important for upskilling both the national and international workforce for this new role.

## Conclusion

More than half the pharmacists who participated were interested, and three-quarters perceived that they felt prepared to work as on-site aged care pharmacists. The majority of pharmacists agreed that they felt prepared to work in both resident and system-level roles, and engage with a variety of stakeholders at all levels of residential aged care to improve medication safety. The findings from this study will be important to residential aged care staff, professional organizations, pharmacy training providers, policy makers and governments in the design and rollout of the on-site aged care pharmacist model in Australia beginning in 2023.

### Supplementary Information

Below is the link to the electronic supplementary material.

Supplementary file1 (DOCX 23 kb)

Supplementary file1 (PNG 1940 kb)
