# Supplementary material for: Pharmacists working in residential aged care: a survey of pharmacist interest and perceived preparedness
Source: Int J Clin Pharm. 2024 Feb 5;46(2):506–14. doi: 10.1007/s11096-023-01686-7 (PMC10960889; doi:10.1007/s11096-023-01686-7)
Supplement: Supplementary file 1 — Supplementary file1 (DOCX 23 kb) [file 11096_2023_1686_MOESM1_ESM.docx]

**Supplementary Table 1: Characteristics of pharmacists and association with INTEREST in working as on-site aged care pharmacist**

| **Characteristic** | Extremely uninterested | Uninterested | Neither interested nor uninterested | Interested | Extremely Interested | Degrees of freedom and test statistic | P-value |
| --- | --- | --- | --- | --- | --- | --- | --- |
| **All participants, n(%)** | 74 (11.5) | 75 (11.7) | 134 (20.8) | 254 (39.5) | 106 (16.5) | NA | NA |
| Age, mean (SD) | 44.6 (10.8) | 46.2 (12.4) | 42.4 (12.0) | 43.0 (13.4) | 43.1 (11.9) | F(4,636) = 1.36 | .248 |
| Years since registering as a pharmacist | 18.9 (12.1) | 23.0 (13.6) | 17.9 (12.4) | 18.2 (13.9) | 16.9 (12.9) | F(4,637)=2.63 | .034 |
| Consultant pharmacist, n(%) |  |  |  |  |  |  |  |
| - Yes | 40 (10.8) | 50 (13.5) | 55 (14.9) | 154 (41.6) | 71 (19.2) | χ2 (4)=22.22 | <.001 |
| - No | 34 (12.5) | 25 (9.2) | 78 (28.8) | 99 (36.5) | 35 (12.9) |  |  |
| Additional Qualification, n(%) |  |  |  |  |  |  |  |
| - Yes | 37 (10.2) | 37 (10.2) | 75 (20.7) | 151 (41.6) | 63 (17.4) | χ2 (4)= 4.123 | .390 |
| - No | 37 (14.2) | 34 (13.0) | 55 (21.1) | 93 (35.6) | 42 (16.1) |  |  |
| Practice Experience^#^, n(%) |  |  |  |  |  |  |  |
| - Community | 65 (11.9) | 61 (11.2) | 105 (19.2) | 221 (40.5) | 94 (17.2) | χ2 (4)= 7.783 | .100 |
| - Hospital | 40 (11.4) | 41 (11.7) | 77 (22.0) | 140 (40.0) | 52 (14.9) | χ2 (4)= 1.785 | .775 |
| - HMR | 26 (8.8) | 42 (14.2) | 47 (15.9) | 118 (39.9) | 63 (21.3) | χ2 (4)= 20.695 | <.001 |
| - Aged Care* | 25 (10.9) | 32 (14.0) | 32 (14.0) | 82 (35.8) | 58 (25.3) | χ2 (4)= 27.878 | <.001 |
| Main Role^#^, n(%) |  |  |  |  |  |  |  |
| - Community | 36 (13.5) | 28 (10.5) | 55 (20.7) | 101 (38.0) | 46 (17.3) | χ2 (4)= 2.576 | .631 |
| - Hospital | 16 (9.2) | 15 (8.7) | 43 (24.9) | 80 (46.2) | 19 (11.0) | χ2 (4)= 11.769 | .019 |
| - HMRs | 11 (9.6) | 17 (14.8) | 17 (14.8) | 43 (37.4) | 27 (23.5) | χ2 (4)= 8.405 | .078 |
| - Aged Care* | 17 (16.0) | 15 (14.2) | 11 (10.3) | 35 (33.0) | 26 (24.5) | χ2 (4)= 15.262 | .004 |

*includes RMMR and/or embedded pharmacist experience, ^#^ not mutually exclusive and p-values derived from binary (yes/no) analyses per line

Abbreviations: HMR, Home Medicines Review; NA, not applicable; RMMR, Residential Medication Management Review; SD, standard deviation.

**Supplementary Table 2: Characteristics of pharmacists and association with PREPAREDNESS to work as on-site aged care pharmacist**

| **Characteristic** | **Strongly disagree or disagree** | **Neither** | **Strongly agree or agree** | Degrees of freedom and test statistic | **p-value** |
| --- | --- | --- | --- | --- | --- |
| **All participants, n(%)** | 56 (8.9) | 98 (15.6) | 475 (75.5) |  | NA |
| Age, mean (SD) | 42.0 (12.0) | 42.6 (12.3) | 43.8 (12.7) | F(2,625)=0.81 | .447 |
| Years since registering as a pharmacist, mean (SD) | 16.9 (12.8) | 17.9 (12.6) | 19.0 (13.5) | F(2,625)=0.76 | .468 |
| Consultant pharmacist, n(%) |  |  |  |  |  |
| - Yes | 15 (4.1) | 44 (12.2) | 303 (83.7) | χ2 (2)= 36.353 | <.001 |
| - No | 41 (15.5) | 54 (20.4) | 170 (64.2) |  | - |
| Additional Qualification, n(%) |  |  |  |  |  |
| - Yes | 31 (8.7) | 53 (14.9) | 271 (76.3) | χ2 (2)= 0.321 | . 852 |
| - No | 22 (8.6) | 41 (16.1) | 192 (75.3) |  | - |
| Practice Experience^#^, n(%) |  |  |  |  |  |
| - Community | 51 (9.6) | 88 (16.5) | 395 (74.0) | χ2 (2)= 4.620 | .099 |
| - Hospital | 22 (6.4) | 42 (12.2) | 280 (81.4) | χ2 (2)= 14.374 | .001 |
| - HMR | 11 (3.8) | 37 (12.7) | 243 (83.5) | χ2 (2)= 23.394 | <.001 |
| - Aged Care* | 8 (3.5) | 20 (8.8) | 198 (87.6) | χ2 (2)= 28.485 | <.001 |
| Main Role^#^, n(%) |  |  |  |  |  |
| - Community | 34 (13.2) | 59 (22.9) | 165 (64.0) | χ2 (2)= 31.637 | <.001 |
| - Hospital | 11 (6.5) | 18 (10.7) | 140 (82.8) | χ2 (2)= 6.733 | .035 |
| - HMRs | 5 (4.4) | 14 (12.4) | 94 (83.2) | χ2 (2)= 5.076 | .079 |
| - Aged Care* | 1 (1.0) | 6 (5.7) | 98 (93.3) | χ2 (2)= 22.121 | <.001 |

*includes RMMR and/or embedded pharmacist experience, ^#^ not mutually exclusive and p-values derived from binary (yes/no) analyses per line.

Abbreviations: HMR, Home Medicines Review; NA, not applicable; RMMR, Residential Medication Management Review; SD, standard deviation.

**Supplementary Table 3: Characteristics of pharmacists and association with preparedness to work as on-site aged care pharmacist based on FORMAL EDUCATION**

| **Characteristic** | **Strongly disagree or disagree*** | **Neither** | **Strongly agree or agree** | Degrees of freedom and test statistic | **P-value** |
| --- | --- | --- | --- | --- | --- |
| **All participants, n(%)** | 174 (27.1) | 124 (19.3) | 345 (53.6) |  | NA |
| Age, mean (SD) | 42.1 (12.3) | 43.6 (12.7) | 44.1 (12.6) | F(2,638)=1.37 | 0.254 |
| Years since registering as a pharmacist, mean (SD) | 17.6 (13.3) | 19.1 (13.0) | 18.9 (13.4) | F(2,639)=0.61 | 0.544 |
| Consultant pharmacist, n(%) |  |  |  |  |  |
| - Yes | 93 (25.1) | 64 (17.3) | 213 (57.6) | χ**^2^** (2)= 5.892 | 0.053 |
| - No | 81 (29.9) | 60 (22.1) | 130 (48.0) |  | - |
| Highest Additional Qualification, n(%) |  |  |  | χ**^2^** (12)= 9.851 | 0.629 |
| - None | 71 (27.2) | 51 (19.5) | 139 (53.3) |  |  |
| - Bachelor/Honors | 16 (28.6) | 12 (21.4) | 28 (50.0) |  |  |
| - Graduate Certificate | 39 (35.1) | 19 (17.1) | 53 (47.7) |  |  |
| - Graduate Diploma | 17 (25.0) | 9 (13.2) | 42 (61.8) |  |  |
| - Masters | 19 (19.8) | 20 (20.8) | 57 (59.4) |  |  |
| - PhD | 8 (25.0) | 7 (21.9) | 17 (53.1) |  |  |

Abbreviations: NA, not applicable; PhD, Doctor of Philosophy; SD, standard deviation.

**Supplementary Table 4: Characteristics of pharmacists and association with preparedness to work as on-site aged care pharmacist based on PRACTICE EXPERIENCE**

| **Characteristic** | **Strongly disagree or disagree** | **Neither** | **Strongly agree or agree** | Degrees of freedom and test statistic | **P-value** |
| --- | --- | --- | --- | --- | --- |
| **All participants, n(%)** | 58 (9.0) | 84 (13.1) | 501 (77.9) |  | NA |
| Age, mean (SD) | 40.7 (11.9) | 42.7 (12.0) | 43.9 (12.6) | F=(2,638)=1.86 | .157 |
| Years since registering as a pharmacist, mean (SD) | 16.0 (12.1) | 16.8 (13.2) | 19.2 (13.4) | F=(2,639)=2.44 | .088 |
| Consultant pharmacist, n(%) |  |  |  |  |  |
| - Yes | 19 (5.1) | 36 (9.7) | 315 (85.1) | χ2 (2)= 28.389 | <.001 |
| - No | 39 (14.4) | 48 (17.7) | 184 (67.9) |  |  |
| Practice Experience^#^, n(%) |  |  |  |  |  |
| - Community | 51 (9.3) | 77 (14.1) | 418 (76.6) | χ2 (2)= 4.260 | .119 |
| - Hospital | 24 (6.9) | 36 (10.3) | 290 (82.9) | χ2 (2)= 10.929 | .004 |
| - HMR | 15 (5.1) | 32 (10.8) | 249 (84.1) | χ2 (2)= 14.342 | .001 |
| - Aged Care* | 8 (3.5) | 21 (9.2) | 200 (87.3) | χ2 (2)= 20.222 | <.001 |
| Main Role^#^, n(%) |  |  |  |  |  |
| - Community | 38 (14.3) | 48 (18.0) | 180 (67.7) | χ2 (2)= 28.676 | <.001 |
| - Hospital | 8 (4.6) | 15 (8.7) | 150 (86.7) | χ2 (2)= 10.914 | .004 |
| - HMRs | 5 (4.3) | 15 (13.0) | 95 (82.6) | χ2 (2)= 3.785 | .151 |
| - Aged Care* | 3 (2.8) | 7 (6.6) | 96 (90.6) | χ2 (2)= 12.053 | .002 |

*includes RMMR and/or embedded pharmacist experience, ^#^ not mutually exclusive and p-values derived from binary (yes/no) analyses per line.

Abbreviations: HMR, Home Medicines Review; NA, not applicable; RMMR, Residential Medication Management Review; SD, standard deviation
