# Supplementary figures and images for: Pharmacists working in residential aged care: a survey of pharmacist interest and perceived preparedness
Source: Int J Clin Pharm. 2024 Feb 5;46(2):506–14. doi: 10.1007/s11096-023-01686-7 (PMC10960889; doi:10.1007/s11096-023-01686-7)

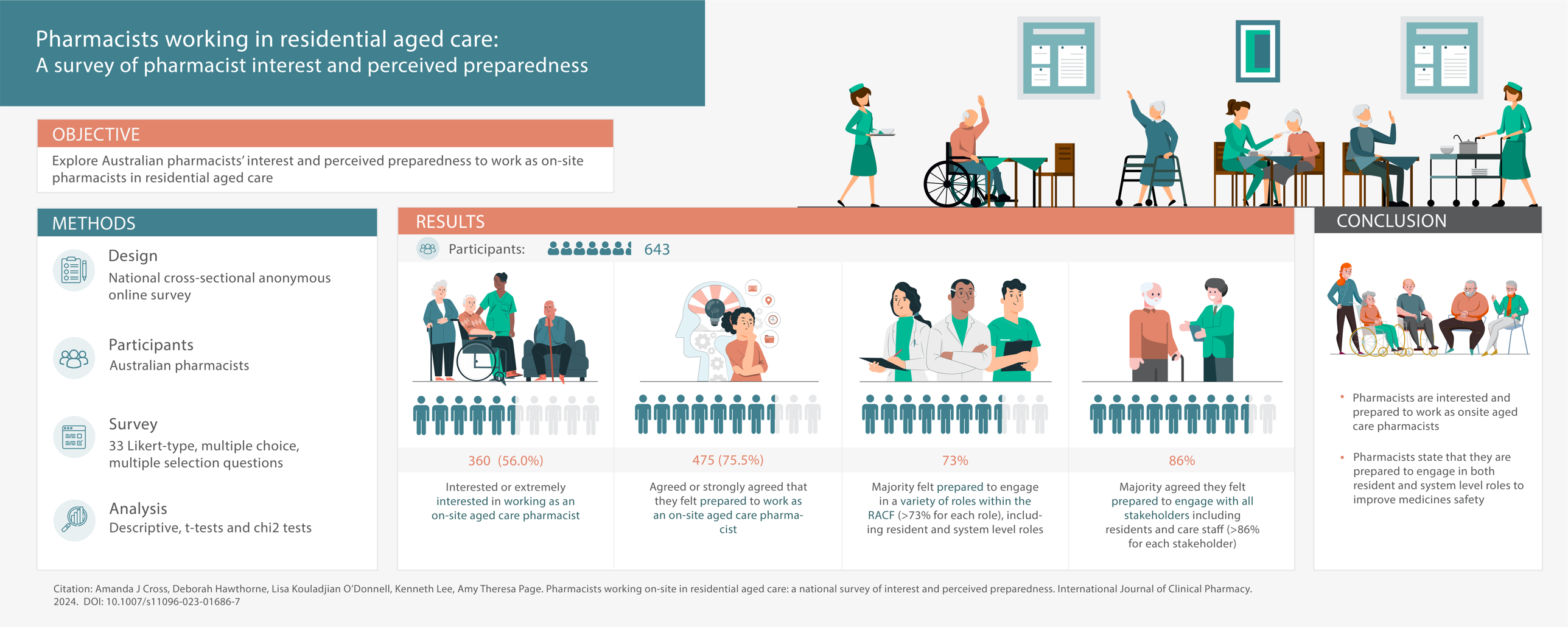

Supplement: Supplementary file 2 — Supplementary file1 (PNG 1940 kb) [file 11096_2023_1686_MOESM2_ESM.png]
